# Endovascular treatment of distal anterior cerebral artery aneurysms using flow modulation devices: mid- and long-term results from a two-center study

**DOI:** 10.3389/fneur.2024.1368612

**Published:** 2024-03-11

**Authors:** Ali Khanafer, Hans Henkes, Jose Cohen, Pablo Albiña-Palmarola, John Moshe Gomori, Michael Forsting, Philipp von Gottberg

**Affiliations:** ^1^Neuroradiologische Klinik, Klinikum Stuttgart, Stuttgart, Germany; ^2^Medizinische Fakultät, Universität Duisburg-Essen, Essen, Germany; ^3^Department of Neurosurgery, Hadassah-Hebrew University Medical Center, Jerusalem, Israel; ^4^Department of Radiology, Hadassah-Hebrew University Medical Center, Jerusalem, Israel

**Keywords:** flow diversion, aneuryms, ACA, pericallosal aneurysm, endovascular treatment

## Abstract

**Purpose:**

Flow-diverter (FD) stents have become an established treatment for intracranial aneurysms in recent years, but their use for aneurysms in distal cerebral vessels with small carrier vessel diameters remains controversial. This study describes the method and mid- and long-term outcomes of FD treatment of distal anterior cerebral artery aneurysms (DACAAs) at two neurointerventional centers, to elucidate this topic and provide more in-depth data.

**Methods:**

Data for all patients at two neurointerventional centers who were treated with FDs for DACAAs in the pericallosal and supracallosal segment of the anterior cerebral artery were retrospectively analyzed. Data on periprocedural complications, and short-, mid- and long-term follow-up findings were recorded.

**Results:**

Forty-one patients were eligible for inclusion in the study. Three FD models were used, one of which had an anti-thrombotic coating. Two periprocedural complications (5%) occurred but did not cause a change in the mRS. In the long-term follow-up, at 29 months and beyond, 83% of assessable patients showed complete occlusion of the aneurysms without new neurological deficits.

**Conclusion:**

FDs are a safe and effective treatment approach for DACAAs. This study indicated a low risk of complications, and high closure rates in short-, mid- and long-term follow-up.

## Purpose

During the past 20 years, flow-diverters (FDs) have emerged as the most important technological innovation for endovascular treatment (EVT) of intracranial aneurysms (1). FD implantation has recently become one of the most widely used methods for the treatment of intracranial aneurysms (2). These devices redirect arterial blood flow through the parent vessel, thereby leading to flow stasis and occlusion of the aneurysm. Results from several recent publications have highlighted the reliability and efficacy of FD treatment (3–5).

Most studies have reported the use of FDs to treat aneurysms in proximal cerebral vessels, including the internal carotid artery (ICA), anterior communicating artery (AcomA), and middle cerebral artery (MCA). The use of FDs to treat distal intracranial aneurysms, notably distal anterior cerebral artery aneurysms (DACAAs), such as those in the pericallosal artery (PA), remains debated (6–8) because of the high rates of peri- and post-procedural complications, and morbidity and mortality resulting from the narrow diameters of the parent vessels of these distal segments.

In the present study, we report the results from two centers in which FDs were used for EVT of DACAAs.

## Methods

### Patient population

In this retrospective study, we report data from two high-volume neurointerventional institutes. We analyzed all DACAA cases treated with FDs between August 2011 and November 2022. We focused specifically on the treatment of aneurysms distal to the AcomA, specifically in the pericallosal and supracallosal segments of the anterior cerebral artery (ACA). We included only cases in which DACAAs were treated exclusively with FD implantation, either as an initial treatment or a follow-up treatment after reperfusion. AcomA aneurysms were not included in this study.

Demographic data; anatomic characteristics; pre- and postprocedural complications; and clinical and angiographic findings from the last available follow-up were recorded for all patients.

### Endovascular treatment

All treatments were performed in patients under general anesthesia with a 6 F guiding catheter or 8 F guiding catheter with a 5 F intermediate catheter in cases of severe vessel elongation, with guidance provided by bi-plane digital subtraction angiography (DSA) (Axiom Artis, Siemens, Erlangen, Germany; Azurion, Philips, Eindhoven). The standard approach included femoral access using short sheaths, predominantly on the right side. All FDs were deployed via Trevo Pro 18, Excelsior XT27 (Stryker Neurovascular, Kalamazoo, MI, United States), Headway 21 (MicroVention Terumo, Tokyo, Japan) or Prowler Select Plus (Cerenovus, Johnson & Johnson, New Brunswick, NJ, United States) microcatheters.

All patients undergoing FD procedures received intravenous administration of 3,000–5,000 IU heparin. Heparinized irrigation solutions were used in all catheters (5,000 IU unfractionated heparin/L). FD implantation was performed by the two most experienced interventionalists at each center.

### Deployed FDs

The following types and models of FD were deployed: (1) Silk Vista Baby (Balt Extrusion), (2) Pipeline Embolization Device (PED) (Medtronic), and (3) p64/p64 MW HPC/p48 MW/p48 MW HPC (phenox).

Data were collected over a 12 years period. Most treatments occurred in the final third of this period. Specifically, two EVT treatments from 2011 to 2014, 11 EVT treatments from 2015 to 2018, and 28 EVT treatments from 2019 to 2022 were performed. Not all included FD models were available in each period. During each temporal period, the integrated FD models were not available. Therefore, in each temporal period, attempts were made to use FDs with thinner profiles, or monoplatelet inhibition instead of dual platelet inhibition. The most commonly used FD (p48 MW HPC) in this study, and the only FD that can be introduced in a 0.017″ microcatheter (Silk Vista Baby), were available only in the final third of the 12 years period. p64 and p48 MW were used exclusively in the first 8 years.

FD length and diameter were selected on the basis of 2D- and 3D-calibrated measurements of the distance between the proximal and distal landing zones; the size of the aneurysm neck; and the diameter of the target vessel. Measurements were performed either periprocedurally or during earlier DSA examinations. Measurements were performed on 2D images with maximum zoom in two positions, usually in 90° posterior–anterior and lateral projection, and 45° left and right anterior oblique projection, with bi-planar DSA. If 3D images were available, additional measurements were taken. If the carrier vessel was less than 2 mm thick, the thinnest available FD was selected, such as the p48 HPC MW (2 mm) or Silk Vista Baby (2.25 mm). For larger carrier vessels, the FD size was increased to the next larger diameter. The choice of the required diameter depended primarily on the availability of thin FDs on the market; such FDs were unavailable in the first years of the study. No measurement software was used for the selection of FDs.

### Medications

Patients to be implanted with p64 HPC or p48 HPC FDs received single antiplatelet therapy (SAPT) with either 1 × 10 mg prasugrel *per os* (PO) daily for at least 5 days before treatment or a loading dose of 30 mg prasugrel 24 h before the procedure.

Patients receiving uncoated FDs underwent dual antiplatelet therapy (DAPT) including 1 × 100 mg acetylsalicylic acid (ASA) PO daily and 2 × 90 mg ticagrelor PO; 1 × 10 mg prasugrel PO; or 1 × 75 mg clopidogrel PO daily for at least 5 days before treatment. Other patients received a loading dose of 500 mg ASA and 600 mg clopidogrel; 180 mg ticagrelor; or 30 mg prasugrel 24 h before the procedure. A multiplate analyzer (Roche Diagnostics, Mannheim, Germany) and VerifyNow (Accriva, San Diego, CA, United States) were used to assess the sufficiency of platelet inhibition on the day of the intervention and at regular intervals after FD implantation. If the responses to the aforementioned APT regimens were too low or excessive, the doses were modified to twice or half the standard dose, respectively. Patients to be implanted with a p64 HPC or p48 HPC FDs received 1 × 10 mg prasugrel PO daily for 6 months; this regimen was then changed to 1 × 100 mg ASA PO daily. The postprocedural medication of the patients implanted with uncoated FDs included 100 mg ASA PO, to be continued indefinitely, and 1 × 75 mg clopidogrel, 2 × 90 mg ticagrelor or 1 × 10 mg prasugrel PO daily for at least 1 year.

For the long-term treatment, all patients were advised SAPT with 100 mg ASA PO daily lifelong.

### Data collection and follow-up

Patients were scheduled for clinical and angiographic follow-up examinations as follows: early follow-up at 3–9 months; mid-term follow-up at 12–24 months; and long-term follow-up after 24 months. Assessment of aneurysmal occlusion was recorded according to the O’Kelly Marotta (OKM) scale, on the basis of the degree of aneurysm perfusion (9). Adequate occlusion was defined as OKM grades C or D.

Neurological examinations were performed by a neurologist or a certified stroke nurse within ≤24 h (periprocedural period) as well as during the postprocedural period (>24 h and ≤30 days) and the follow-up (>30 days), according to the modified Rankin Scale (mRS) (10). Morbidity was defined as any permanent deterioration with respect to the baseline mRS.

### Ethical standards

All patients or their legal representatives provided written informed consent at least 1 day before the procedure, after having been informed of the planned treatment strategy and possible complications. The participants consented to data collection, analysis and anonymous publication of their findings (IRB F-2018-110).

The relevant ethics committee was consulted locally by each center.

### Statistical analysis

Continuous data are described as the mean, median, minimum, and maximum. Hazard ratios with 95% confidence intervals (CIs) were estimated with Cox regression to analyze the influence of aneurysm location on complication probability. Numbers and percentages were used to describe categorical data. Stata/IC 16.1 for Unix was used for the statistical analysis.

## Results

### Location

**Figure 1:**
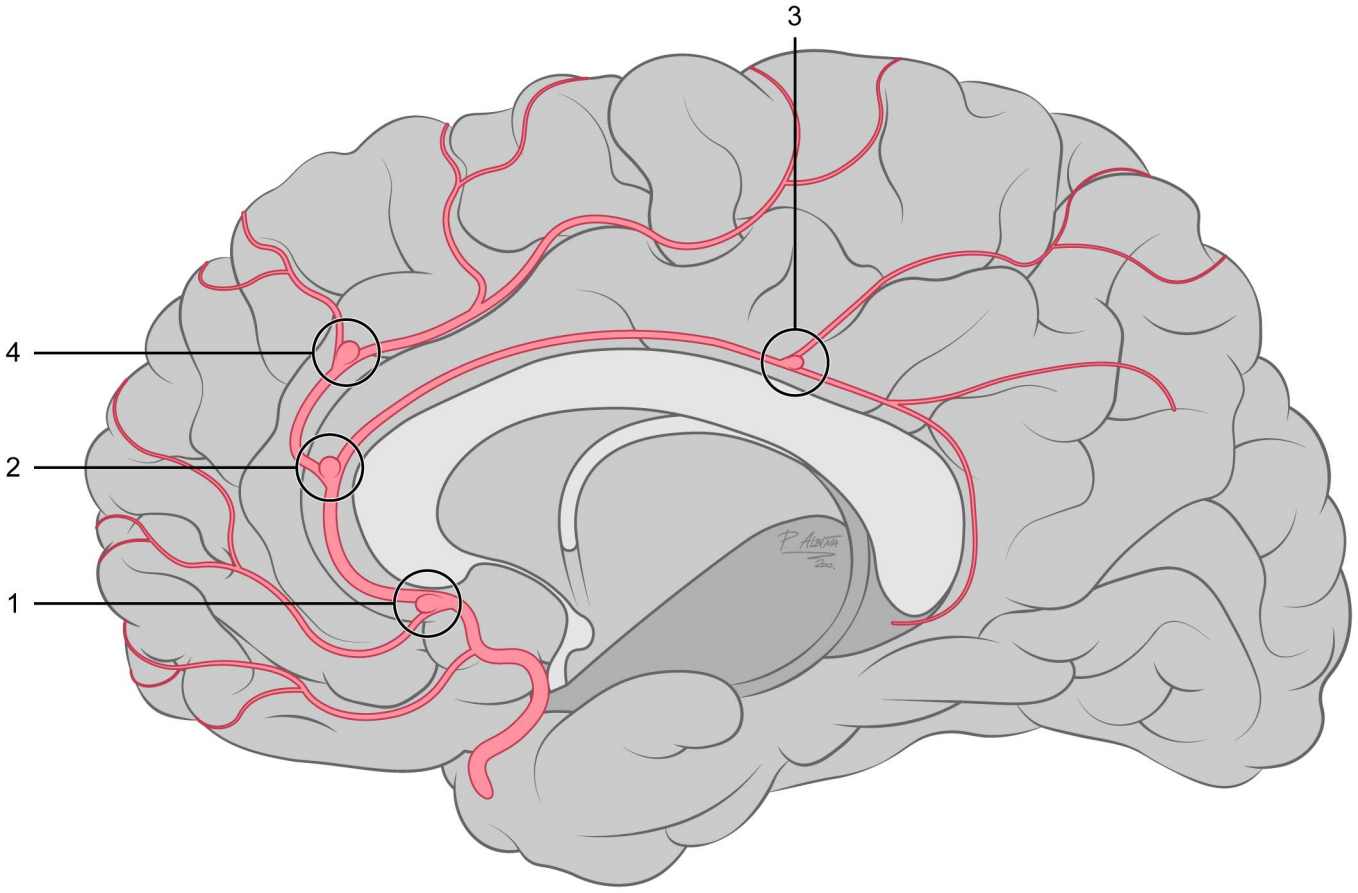
Schematic representing the locations of DACAAs in the 41 patients enrolled in this study.

The topographic distribution of the DACAAs is shown in Figure 1. The DACAAs treated with FDs in this study were located at (1) the branch point of the ACA into the frontopolar artery; (2) the division point of the ACA into the PA and callosomarginal artery (CMA); the branch point of the PA into the superior parietal artery (3); and (4) the branch point of the CMA into the anterior internal frontal artery.

### Patients and aneurysm characteristics

**Figure 2:**
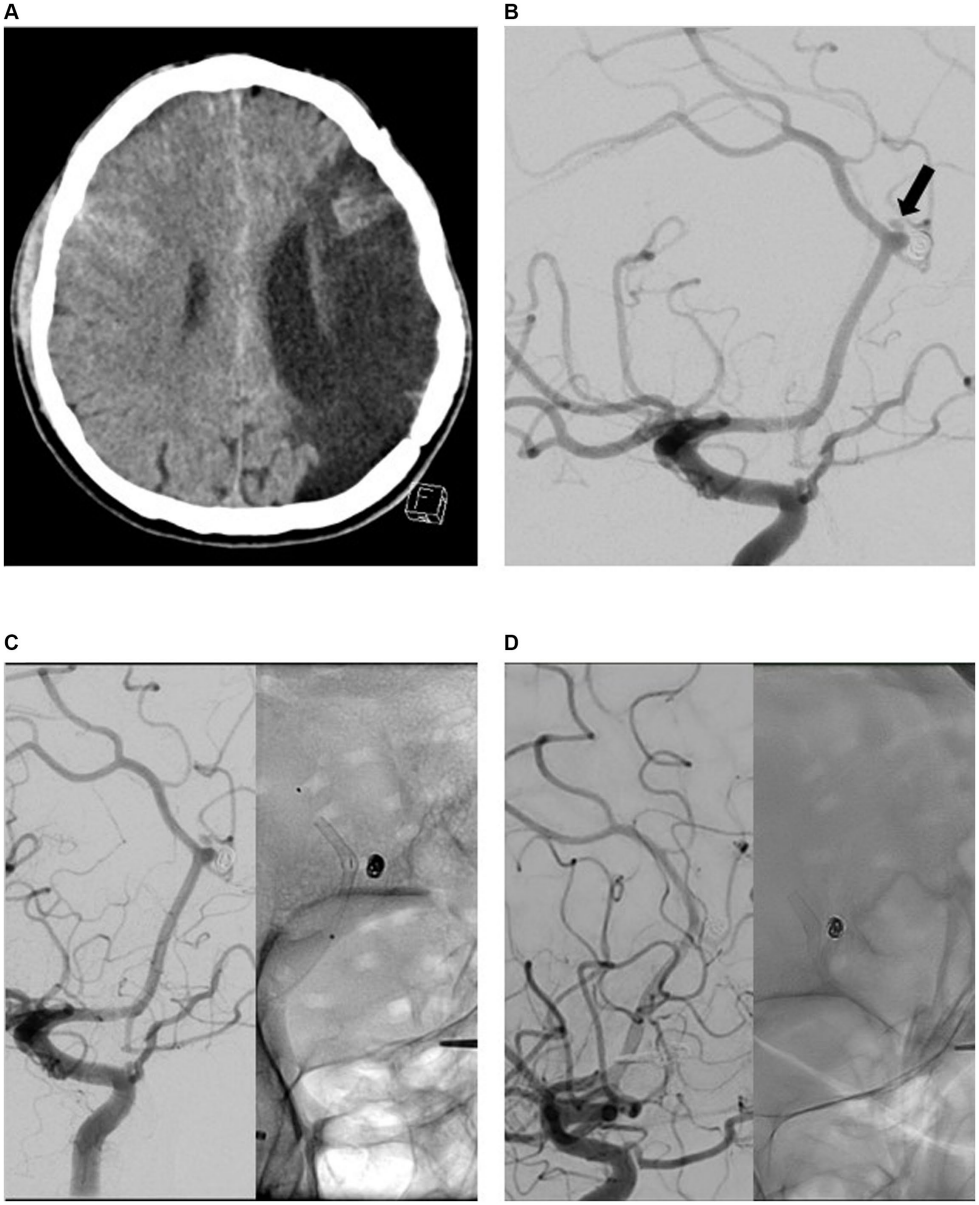
Endovascular treatment (EVT) and diagnostic imaging in a patient with aneurysmal subarachnoid hemorrhage (aSAH) due to rupture of a recurrently perfused distal anterior cerebral artery aneurysm (DACAA) that occurred 15 years after coil occlusion. The primary lesion was evident on computed tomography imaging **(A)**. Digital subtraction angiography (DSA) with contrast-medium injection into the right internal carotid artery (ICA; lateral view 45°) confirmed reperfusion of a formerly coiled DACAA (at arrow) **(B)**. Final DSA imaging with contrast-medium injection into the right ICA during EVT with flow diverter (FD) implantation (p48 MW HPC) **(C)**. The first DSA follow-up after 3 months revealed complete occlusion of the aneurysm **(D)**.

Forty-one patients diagnosed with DACAAs were treated with FD implants between August 2011 and November 2022 (Table 1). The cohort included 33 females (80%). The mean age of all patients enrolled in the study was 56 years (range 6–81 years). The mean DACAA neck size was 2.31 mm (range 1–6 mm); seven (17%) cases were below 2 mm at maximum diameter, 22 (54%) cases were 2–4 mm, and 11 (27%) cases had a maximum diameter of 4–6 mm (See Figure 2).

**Table 1:** Study population demographics and description of the aneurysms and FDs.

Patients	
Number of Patients/Aneurysms	41/41
Female/Male	33/8
Age	Mean 56 years (range 6–81 years)
Aneurysm/FD	
Aneurysm neck size	Mean 2.3 mm (range 1–6 mm)
Aneurysm diameter	Mean 3.3 × 2.8 (range 1 × 1–6 × 5 mm)
Pretreated (coiling)	29% (*n* = 12)
Diameter proximal/distal landing zone	2.0 mm/1.7 mm (range 0.9–2.8 mm/0.9–2.5 mm)
Difference proximal/distal; landing zone	0.25 mm (0–0.8 mm)
p64 HPC	1
p48 HPC	19
p64	7
p48	5
Silk Vista Baby	7
Pipeline	2
Original diameter of the selected FD before implantation	Mean 2.3 mm (range 2–3.5 mm)
Original length of the selected FD before implantation	Mean 14 mm (range 9–20 mm)

Only one FD was implanted per aneurysm. FD implantation was the first treatment for 27 of the aneurysms (66%); in the remaining 14 cases (34%), FDs were used to treat remnants or recurrences after endovascular pretreatment (coil occlusion, *n* = 12). FD implantation was performed in two cases of acute subarachnoid hemorrhage after rupture of the aneurysm.

Aneurysms were located proximal to the PA bifurcation in 7% (distal A2-segment, *n* = 3), at the bifurcation of the PA in 73% (*n* = 30) and distal to the PA bifurcation in 20% (*n* = 8) of the cases evaluated in this study.

The mean diameters of the distal and proximal landing zones were 1.7 mm (range 0.9–2.5 mm) and 2.0 mm (range 0.9–2.8 mm), respectively. The mean difference in diameters between the proximal and distal landing zones was 0.25 mm (range 0–0.8 mm).

Nine of the implanted FDs (seven p64 classic and two Pipeline) could be introduced only with a 0.027 microcatheter. Probing the DACAA carrier vessel was more challenging with the 0.027 microcatheter than the 0.021 and 0.017 microcatheters. In four of nine cases, because the 0.027 microcatheter could not be directly introduced into segment A3, a thinner microcatheter was used to access the carrier vessel; subsequently, an exchange maneuver with a 300 cm microwire was performed. This process increased the procedure time, radiation time and material use. The documented periprocedural complications occurred after EVTs with 0.027 microcatheters, but we observed no evidence of a direct correlation. No difference in probing or device delivery was observed between the 0.017 and 0.021 microcatheters.

### Angiographic follow-up

Early follow-up (FU1, 3–9 months) was performed at a median of 146 days post-procedure for all 41 patients (i.e., 41 aneurysms). Complete aneurysm occlusion (OKM D) was observed in 31 (76%) cases. Neck remnants (OKM C) were detected in two (5%) aneurysms. Two cases (5%) exhibited subtotal aneurysmal filling (OKM B), and six (15%) remained unchanged (OKM A). Mild intimal hyperplasia without stenosis was observed in two patients (5%). All 41 patients were asymptomatic at that time.

Mid-term follow-up (FU2, 10–18 months) was performed for 34/41 (83%) of the patients at a median of 340 days. Seven patients who were not scheduled for follow-up at that time had aneurysms graded OKM D at their earlier DSA examination.

Mid-term follow-up DSA revealed complete aneurysm occlusion (OKM D) in 25 of the 34 (74%) patients. One patient’s aneurysm shifted to OKM D from OKM B. Thus, 32 of the original 41 patients (78%) had grade OKM D aneurysms when the seven patients identified as having this grade in FU1 were included. One patient’s aneurysm shifted from OKM B to OKM C, thus resulting in a total of three patients (9%) at this grade. Sac remnants (OKM B) were observed in three aneurysms (9%) that were graded OKM A at FU1. Aneurysms from three of the original six patients remained at OKM A (9%). All 34 patients reviewed at FU2 remained asymptomatic. The two patients who presented with mild intimal hyperplasia at FU1 also showed no change in grade; no additional patients presented with intimal hyperplasia at that time.

Long-term follow-up (FU3, 19–28 months) was performed after a median of 613 days for 22 of the original 41 patients (54%). Sixteen patients were not scheduled for follow-up because their aneurysms were graded OKM D in FU1 and/or FU2; three patients did not appear for scheduled examinations and were not followed up. At FU3, 16 patients with aneurysms graded at OKM D (73%) showed no additional shifts in the scale. Two patients originally diagnosed with neck remnants (OKM C) remained in this condition and showed no changes with respect to former follow-ups. Sac remnants (OKM B) were observed in aneurysms from three patients (14%), including one that shifted from OKM A to OKM B during this interval. Of note, one patient whose aneurysm was formerly graded OKM D presented with reperfusion and conversion to OKM A (5%). This patient underwent a second procedure with an FD device, because the results from the first implantation procedure were considered insufficient. Therefore, we assumed that 31 of the total 41 aneurysms (76%) were graded at OKM D. Intimal hyperplasia improved in the two reported cases; no new in-stent stenosis or intimal hyperplasia developed in any patients in this study. All patients evaluated at FU3 remained asymptomatic.

A final follow-up (FU4, >29 months) was performed after a median of 1,489 days for nine of the original 41 patients (22%). Eighteen patients whose aneurysms were graded OKM C or D at an earlier FU were not scheduled for FU4. Five patients dropped out of the study because of a need for re-treatment, and nine patients were lost to follow-up. At FU4, aneurysms from eight of the nine patients were graded OKM D (89%). One patient’s aneurysm scored as OKM C at an earlier FU and was graded OKM D. Thus, at that time, the aneurysms from 34 of the 41 patients were graded OKM D (83%). One aneurysm with a neck remnant (OKM C) remained unchanged (11%). None of the aneurysms had scores of OKM B or OKM A at that time, and no in-stent stenosis or intimal hyperplasia was observed. All patients evaluated at FU4 were asymptomatic.

The changes in OKM grades at each follow-up visit are summarized in Table 2.

**Table 2:** OKM grade at follow-ups (FU).

	*n*	OKM D (*n*; change)	OKM C (*n*; change)	OKM B (*n*; change)	OKM A (*n*; change)
FU1 (3-9 m)	41, pop. 100%	76% (31; 0)	5% (2; 0)	5% (2; 0)	15% (6; 0)
FU2 (10-18 m)	34, pop. 83%	78% (25;+1 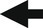 B)	9% (3; +1 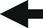 B)	9% (3; +3 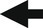 A)	9% (3; −3)
FU3 (19-28 mo)	22, pop. 54%	76% (16; −1)	9% (2; 0)	9% (2; +1 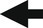 A)	14 (3; +1 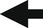 D)
FU4 (>29 mo)	9, pop. 22%	83% (8;+1 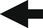 C)	11% (1; 0)	0 (0)	0 (0)
Latest possible follow-up	41	83% (34)	7,3% (3)	7,3% (3)	2,4% (1)

The arrows indicate changes in OKM grades and the number of patients involved with respect to each previous follow-up time point.

**Figure 3:**
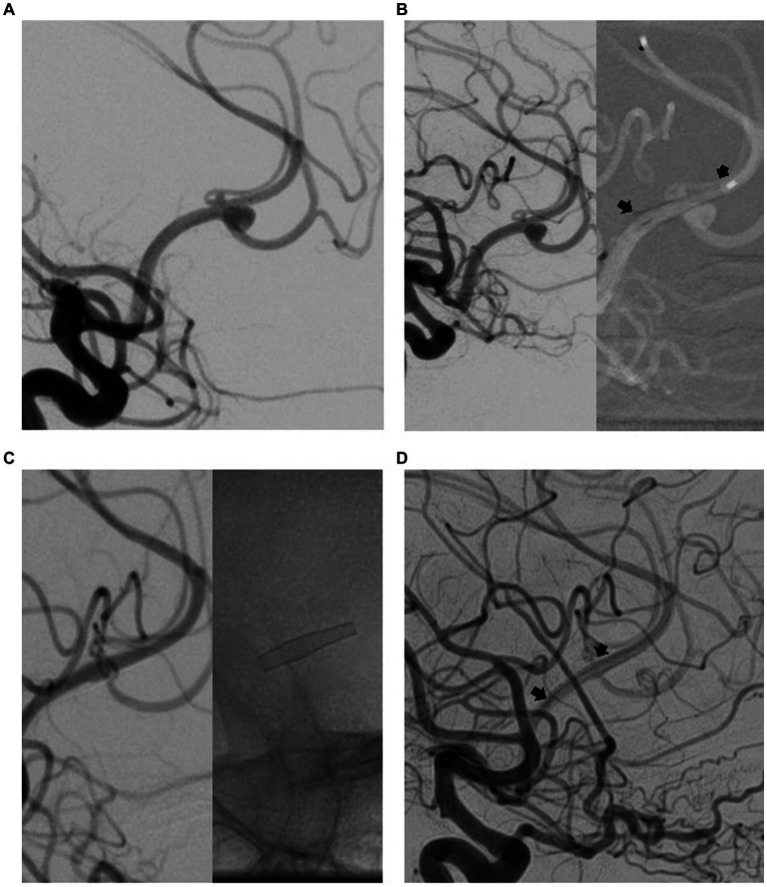
Endovascular treatment (EVT) and diagnostic imaging in a patient with distal anterior cerebral artery aneurysm (DACAA). Digital subtraction angiography (DSA) with contrast-medium injection of the right internal carotid artery (ICA; lateral projection) revealed a DACAA at the division of the anterior cerebral artery (ACA) into the pericallosal artery (PA) and callosomarginal artery (CMA) **(A)**. Final DSA with contrast medium injected into the right ICA demonstrated the effects of EVT implantation of a flow diverter (FD) (p48 MW HPC; between arrowheads) **(B)**. The first DSA follow-up (FU) after 3 months indicated complete occlusion of the aneurysm **(C)**. The aneurysm remained completely occluded at 12 months FU **(D)**.

All patients with aneurysms scored at OKM A or OKM B in long-term follow-up (FU3) were advised to undergo re-treatment and were not included in the final follow-up (FU4). An exemplary case is shown in Figure 3.

In these patients, if consented, a second FD was implanted in the carrier vessel under the aneurysm, resulting in sufficient reduction of blood in stream into the aneurysm or complete occlusion within the 6 subsequent months (OKM C or D).

The differences in results among the implanted FDs are shown in Table 3.

**Table 3:** Comparison of FD results in relation to occlusion rates, technical difficulties, and peri- and post-procedural complications.

	p48 HPC	p64	Silk Vista Baby	p48	Pipeline	p64 HPC
Number	19	7	7	5	2	1
OKM C + D at FU 3–9 m	84.2% (16)	85.7% (6)	100% (7)	40% (2)	100% (2)	100% (1)
OKM C + D Latest FU	89.5% (17)	85.7% (6)	100% (7)	80% (4)	100% (2)	100% (1)
Need for a device for better wall adaptation	0	0	0	0	0	0
Intimal hyperplasia	10.5% (2)	0	0	0	0	0
Silent post-procedural emboli	36.8% (7)	15.8% (3)	15.7% (3)	40% (2)	0	0
Ischemia	0	0	0	0	50% (1)	0
Side branch occlusion	5.2% (1)	28.5% (2)	14.2% (1)	0	50% (1)	0

### Periprocedural complications

**Figure 4:**
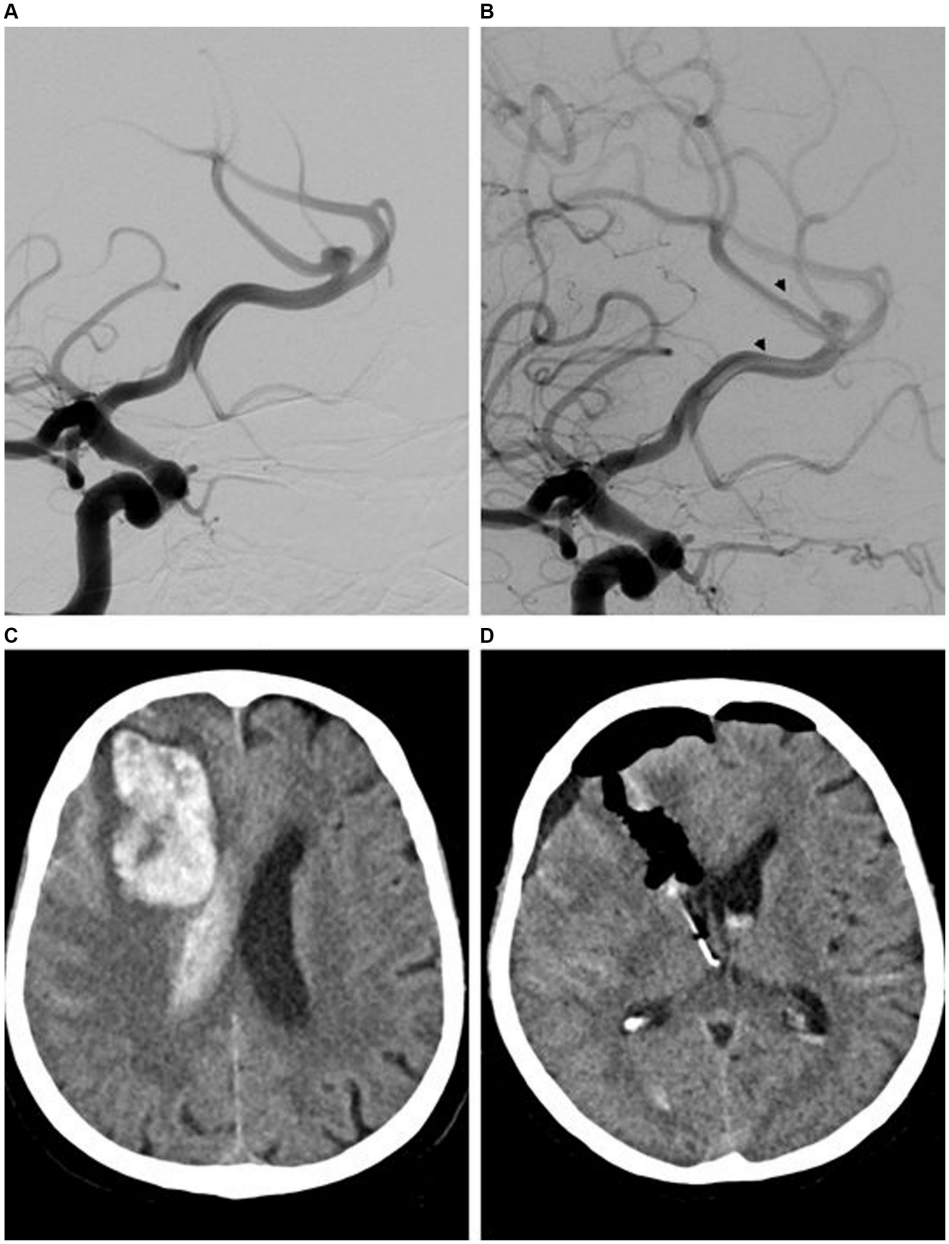
Endovascular treatment (EVT) and diagnostic imaging in a patient with a distal anterior cerebral artery aneurysm (DACAA) and periprocedural intracerebral hematoma. Digital subtraction angiography (DSA) with contrast-medium injection of the right carotid artery (ICA; lateral view 45°) revealed a DACAA at the division of the anterior cerebral artery (ACA) into the pericallosal artery (PA) and callosomarginal artery (CMA) **(A)**. A final DSA with contrast medium injected into the right ICA highlighted cerebral circulation after implantation of the flow diverter (FD) (p64, between arrowheads) **(B)**. The patient presented with headache and diminished vigilance on the evening of the treatment day. The subsequent computed tomography (CT) examination revealed a large parenchymal hematoma in the right frontal lobe believed to be due to hyperresponsiveness to the DAPT (ASA and ticagrelor), as confirmed by Multiplate (ADP 10 U, ASPI 11 U) and VerifyNow (P2Y12 8/255; 97%, ARU 444) analysis **(C)**. A follow-up CT was performed after craniotomy with evacuation of the hematoma and placement of an external ventricular drain on the right frontal side **(D)**. The patient was discharged with improved neurological status after hospitalization (mRS 2). After rehabilitation, she returned to her baseline neurologic status (mRS 0).

Two of the 41 patients (5%) experienced periprocedural complications (24 h after the start of the intervention). One patient developed an intracranial hematoma, probably because of hyper responsiveness to the APT. After surgical treatment and rehabilitation, her neurological status improved to her baseline status of mRS 0 (Figure 4). One patient developed minor ischemia in the ACA territory 8 h after FD implant deployment, which manifested as slightly impaired movement of the contralateral leg. However, by the time of discharge, he had recovered to his baseline mRS of 0. One patient developed a dissection of the ICA during diagnostic runs performed before treatment and was treated by implantation of two flow-diverter stents. The patient developed no neurological deficits associated with the incident. Because this complication was not directly associated with the DACAA treatment, it was not evaluated as an intra- or periprocedural complication in this study.

On pre-discharge magnetic resonance imaging (MRI) scans, silent ischemic microemboli were detected on diffusion-weighted imaging examinations in 12 of 41 patients (29%). However, with the exception of the patient with ACA-associated ischemia discussed above, the remaining 11 patients were asymptomatic on neurological examination. No embolic complications were reported during the procedures.

### Postprocedural complications in clinical follow-up (24 hours–30 days)

The patients experienced no complications in the immediate postprocedural phase, and no complications or clinical deterioration during the follow-up period.

### Statistical analysis

The statistical evaluation with regard to possible correlations between the localization of the aneurysm and a complication, the probability of achieving OKM D in the first follow-up in relation to an FD type or the localization did not yield any reliable results and is therefore not presented further.

**Figure 5:**
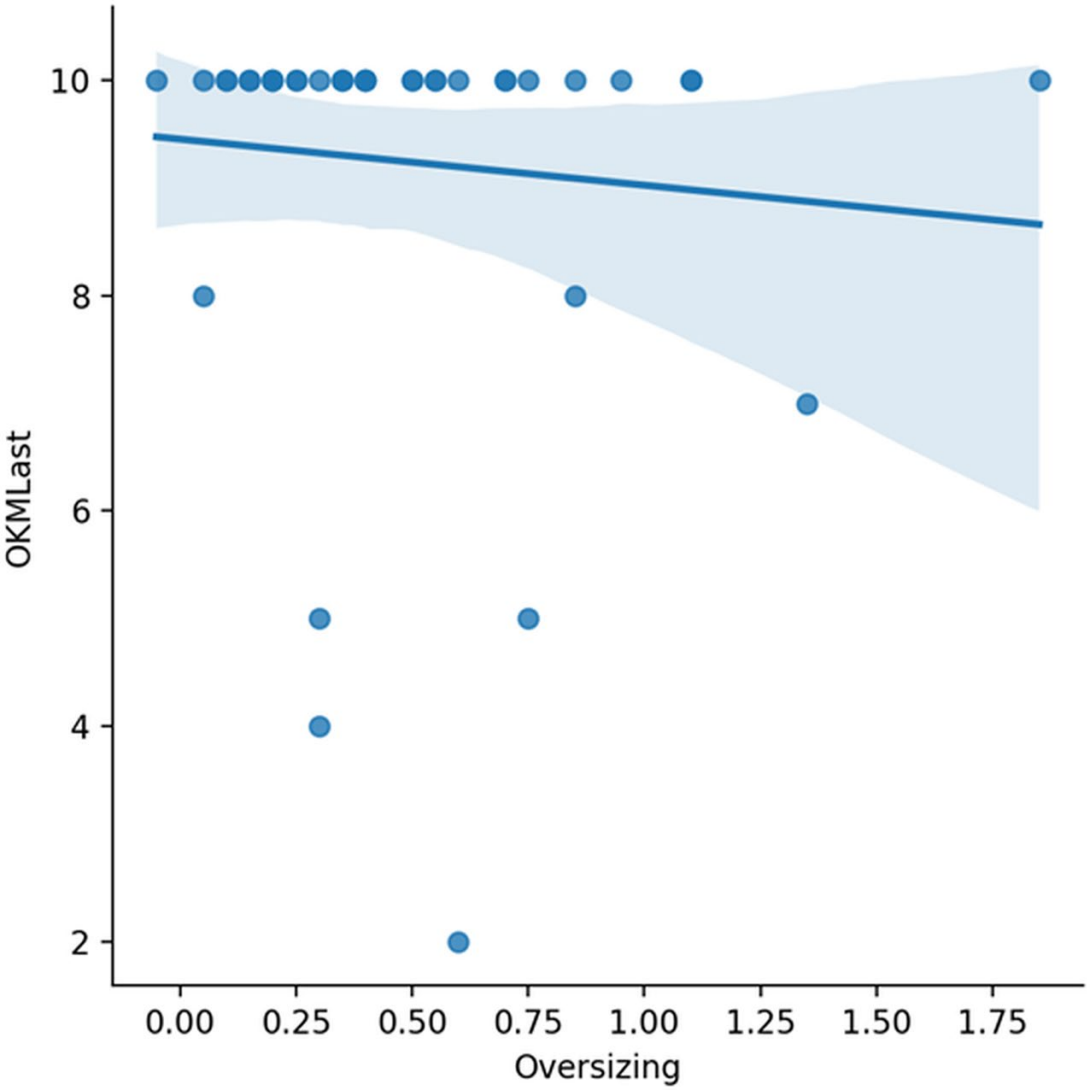
A higher degree of oversizing (*x*-axis in mm) was in lightly decreasing linear relationship to OKM 10 in the last follow-up.

In the evaluation of the degree of oversizing in relation to closure success, there was a linear descending relationship between the degree of oversizing and the OKM D result in the last follow-up (Figure 5).

## Discussion

This article reports the results for 41 patients with DACAAs who underwent treatment at one of two high-volume neurointerventional centers. DACAA is a rare finding representing <5% of all intracranial aneurysms (11, 12). Patients diagnosed with DACAAs have an elevated probability of having multiple intracerebral aneurysms. For example, the dual diagnosis of MCA and AcomA aneurysms has been reported to have an incidence of approximately 38–46% (13, 14). Most DACAAs are located at the bifurcation of the PA and the CMA at the genu of the corpus callosum. DACAAs are primarily small saccular-type aneurysms with relatively wide necks (15); their relatively small size may be due to their tendency to rupture before becoming large (16), potentially because of the diminished resistance of the arachnoid at the level of the pericallosal cisterns (17). Alternatively, larger DACAA lesions may tend to rupture because of the reduced wall stress experienced by vessels with a narrow diameter, such as the distal A2 (i.e., a pericallosal) branch. This finding may also explain why the vessel wall of the PA is less likely to sag than that of the distal ACA or MCA.

DACAA rupture is frequently associated with intracerebral hematoma in the frontal lobe and may be localized to either side (15). In a study by Khanafer et al. (18), 50 of 1,064 (4.7%) cases of subarachnoid hemorrhage due to aneurysm rupture were found to directly result from ruptured DACAAs. In this subgroup, 15 of the patients (30%) were symptomatic because of vasospasm (18). Given the elevated likelihood of rupture and bleeding, treatment is always recommended for incidental DACAAs, despite their small size.

In the 2000s and early 2010s, incidental and ruptured DACAAs were treated almost exclusively with one or more surgical approaches (15, 19–22). However, DACAA surgical clipping may be considered more challenging than the clipping of proximal aneurysms of the anterior cerebral artery. DACAAs can be difficult to access surgically and require detailed knowledge of the microsurgical anatomy. Furthermore, owing to the small size and thin wall of the distal ACA and adjacent small vessels, the risk of vascular dissection is relatively high (15, 19). Fusiform and dissecting DACAAs present additional challenges for surgical treatment and, in some cases, require more complex approaches, including wrapping, proximal occlusion, excision, trapping, parent artery occlusion with preoperative bypass and reconstruction (19).

With the development of interventional neuroradiology, coiling has become a feasible alternative to surgical clipping, particularly after aneurysm rupture (20). To date, only two published studies have compared the outcomes of coiling and clipping as primary treatments for DACAAs. Whereas Suzuki et al. (23) reported no significant difference between outcomes when using these techniques to treat 68 patients (55 treated with clipping), Park et al. (24) reported more favorable clinical outcomes and fewer periprocedural complications in patients treated with endovascular coiling (*n* = 38) rather than surgical clipping (*n* = 46). The frequent small sizes and wide bases of DACAAs can make both surgical clipping and endovascular coiling difficult, and stent-assisted coiling techniques may be required in some constellations and anatomies (A, B).

Adeeb et al. (23) have reported positive outcomes for treating bifurcation aneurysms at different locations. Notably, the occlusion rates for ACA bifurcation were lower (71.4%) than those for basilar tip (91.6%) and ICA (96.7%) bifurcation (C). In a study by Zimmer (24), the WEB 17 was also used as a treatment option for A2 aneurysms in 13 of 44 cases. In this study, three periprocedural complications or difficulties were observed, two of which were A2 aneurysms (D).

FD implantation as a treatment for aneurysms has undergone a remarkable evolution and currently is essential in current neurovascular therapy. In the past 5 years, several case series or single-center studies focusing on the use of FD for DACAAs have been reported (8, 25–31). Nonetheless, the use of FD implantation procedures for the treatment of distal aneurysms remains controversial, because of limited experience and the lack of large treatment studies.

In this study, we observed occlusion rates of 76, 78, and 83% in DSA studies performed at early (3 to 9 months), mid-term (10 to 20 months), and long-term (28 months) follow-up, respectively. The number of occluded aneurysms might be even higher than reported, because several patients with occluded (OKM D) or near-occluded (OKM C) aneurysms were not re-scheduled for ongoing follow-up and/or did not undergo the recommended diagnostic imaging procedures. Nonetheless, these findings are consistent with results presented in previous studies. For example, Lin et al. (25) have reported the results from a study of 28 patients with aneurysms of the distal anterior circulation (including eight in the ACA) who were treated with a PED. Neurovascular follow-up in 27 patients revealed that 21 of the aneurysms (78%) were completely occluded. Similarly, Martínez-Galdámez et al. (30) studied 25 patients who presented with anterior circulation aneurysms and underwent treatment with PED. Angiograms performed 6 months later revealed complete occlusion in 14 cases (64%). Pistocchi et al. (31) have described a series of 30 aneurysms (in 26 patients) that had been treated with a Silk or a pipeline flow diverter, and reported that 79% of the aneurysms were completely occluded at follow-up (mean 13 months). Finally, Dabus et al. (8) have described 20 patients with complex ACA aneurysms, six of which were A2-pericallosal. In that series, 69 and 75% showed complete or complete or near-complete occlusion, respectively.

Although the authors reported no significant differences in occlusion rates between FD implantation and other strategies, the case numbers were too small to enable valid assessment of the various devices.

Regarding complications, the distal position of the implanted FD may be associated with the rate of ischemic complications. The overall rate of complications, i.e. hemorrhage and clinically manifest ischemia, in our study was 5% (silent DWI-lesion not respected), a percentage slightly lower than reported in other similar studies. For example, Pistocchi et al. (31) have reported an overall rate of neurological complications of 11.1%, including 7.4% that were reversible and 3.7% that were permanent. In a study by Lin et al. (32), 10.7% of patients experienced periprocedural complications (i.e., within <30 days). Likewise, in a study by Dabus et al. (8), 10% of the patients experienced at least one neurological event, and 5% developed large intraparenchymal hemorrhages.

Only two of the treated aneurysms showed mild, hemodynamically irrelevant intimal hyperplasia at follow-up. Both patients were treated with a p48 MW HPC FD under SAPT. The two follow-up examinations occurred after 6 months. One of the two patients had changed his medication from prasugrel to ASA several days before the examination, and the second patient was still taking prasugrel. Both patients underwent MP and VN, which did not indicate a sufficient ASA effect in the first patient; consequently, the ASA dose was doubled. In the second patient, the medication was not changed after the tests. As a standard measure for intimal hyperplasia without hemodynamic effects, at our centers, we performed the second FU earlier (after 3 instead of 6 months). The findings indicated no deterioration in either patient, and complete regression was documented in the long-term FU.

DACAA treatment with FDs may cause overlap of side branches such as the CMA or frontopolar arteries, thus resulting in occlusion or stenosis of these arteries. Two cases showed complete occlusion of the CMA, and three cases showed complete occlusion of the frontopolar arteries. Six patients had ≥50% stenosis of the vessel lumen. This phenomenon was observed in four of the six FD types, but no significant association was found. All patients were clinically asymptomatic and showed no neurological deficits.

We treated two patients with acutely ruptured aneurysms with FD implantation. These patients developed no hemorrhagic or ischemic complications and experienced no rebleeding. DAPT or SAPT in combination with heparin increases the risk of hemorrhagic peri- or postprocedural complications. Several research groups have reported that hemorrhagic transformation of clinically silent microinfarcts may occur after endovascular procedures and is an important complication of FD implantation (8, 33, 34). In our series, one patient with a hemorrhagic complication, identified several hours after FD implantation, was probably due to an excessive response to ticagrelor. Nonetheless, the patient improved clinically during her hospital stay and was discharged with an mRS score of 2. At the first and final follow-up, her score was mRS 0.

The statistical finding of a linear decrease between oversizing and OCM D at the last follow-up is surprising, but is most likely biased by the small sample size.

There is disagreement in the current literature about APT in the longer term after FD treatment, and there is still no recommendation or guideline. After initial DAPT followed by SAPT, some centers discontinue platelet inhibition completely 1–2 years after FD implantation, while other centers at least maintain SAPT.

Based on insufficient data and our empirical evidence, the current recommendation to all patients included in this study was lifelong SAPT.

DACAA treatment remains a major challenge because of these aneurysms’ infrequent occurrence; anatomical considerations (i.e., small fundus, wide neck, distal location, frequent localization at a bifurcation and small diameter of the parent vessel); and frequency of ICH after rupture, despite advances in microsurgical clipping and endovascular coiling procedures. The recent development of highly flexible, low-profile FDs offers a safe and efficacious option for the treatment of small distal aneurysms. Furthermore, the development of coated FDs with diminished thrombogenicity enables implantation in patients maintained on SAPT with prasugrel, thus potentially increasing the safety margins (34–36).

## Conclusion

FD implantation is an efficacious and safe treatment option for the management of DACAAs. High occlusion rates were achieved at short-, mid- and long-term follow-up, and were accompanied by few clinically meaningful complications.

## Data availability statement

The raw data supporting the conclusions of this article will be made available by the authors, without undue reservation.

## Ethics statement

The studies involving humans were approved by Ethikkommission der Ärztekammer Nord-Württemberg, Stuttgart, Germany. The studies were conducted in accordance with the local legislation and institutional requirements. The participants provided their written informed consent to participate in this study.

## Author contributions

AK: Conceptualization, Data curation, Investigation, Writing – original draft, Writing – review & editing. HH: Conceptualization, Methodology, Project administration, Supervision, Validation, Writing – review & editing. JC: Methodology, Supervision, Validation, Writing – review & editing. AP: Formal analysis, Visualization, Writing – review & editing. JG: Data curation, Investigation, Supervision, Writing – review & editing. MF: Formal analysis, Methodology, Project administration, Supervision, Validation, Writing – review & editing. PG: Conceptualization, Investigation, Writing – original draft, Writing – review & editing.
